# Response of bacterial communities in rubber plantations to different fertilizer treatments

**DOI:** 10.1007/s13205-019-1821-6

**Published:** 2019-07-04

**Authors:** Zhiyang Zhang, Peisong Zhang, Qinghuo Lin, Zhengzao Cha, Wei Luo

**Affiliations:** 1 Institute of Tropical Agriculture and Forestry, Hainan University, Haikou, Hainan China; 2 Rubber Research Institute, Chinese Academy of Tropical Agricultural Sciences, Haikou, Hainan China

**Keywords:** *Hevea brasiliensis* Muell. Arg., Natural rubber yield, Organic fertilizer, Chemical fertilizer, Bacterial communities

## Abstract

In the present study, the effects of chemical fertilizer (CF) and organic fertilizer plus chemical fertilizer application (OF–CF) on natural rubber yield, soil properties, and soil bacterial community were systematically investigated in rubber plantations. The rubber dry yield was 26.3% more in the OF treatment group than in the CF treatment group. The contents of total nitrogen (TN), available nitrogen (AN), available phosphorus (AP), and available potassium (AK) as well as soil organic matter (SOM) and pH value were higher following OF–CF treatment. Using Illumina sequencing, a total of 927 operational taxonomic units (OTUs) were obtained following CF treatment, while 955 OTUs were obtained after OF–CF treatment. Relative abundance analysis showed the relative abundances of four phyla (Acidobacteria, Proteobacteria, Actinobacteria, Gemmatimonadetes) were different between the two treatment groups. Correlation analysis revealed Acidobacteria, Bacteroidetes, Thaumarchaeota, Elusimicrobia, Verrucomicrobia were the key taxa that determined the soil properties. Additionally, five OTUs (OTU_506, OTU_391, OTU_189, OTU_278, OTU_1057) were thought to be related to the biodegradation of natural rubber. Taken together, these results improve our understanding of the OF-mediated improvement in soil fertility and contribute to the identification of rubber-degrading bacteria in rubber plantations.

**Electronic supplementary material:**

The online version of this article (10.1007/s13205-019-1821-6) contains supplementary material, which is available to authorized users.

## Introduction

Chemical fertilizer (CF) significantly increases the yield of crops and has majorly contributed to the green revolution in the twentieth century. However, the mismanagement of inorganic nitrogen and phosphorus inputs is a well-known inefficiency that has posed a threat to the environment (Martínez-Alcántara et al. 2016). Organic fertilizer (OF) has been recently used as substitutes for CF because of they are environmentally friendly. In addition, OF offer an obvious advantage of improving carbon sequestration, pH balance, cation/anion retention, and microbial communities in soil (Zhang et al. 2011). In comparison with CF, OF can regulate soil properties and improve the production of several crops (Liu et al. 2015; Wang et al. 2016a).

Soil microorganisms form complicated microbial communities that regulate the nutrient cycles and influence soil characteristics, plant growth, and ecosystem sustainability (van der Heijden et al. 2008). Bacteria are the most abundant group of soil microorganisms. The determination of soil bacteria is important to understand the bacterial diversity and community composition. Phospholipid fatty acids profiling and 16S rDNA fingerprinting are the key strategies employed for the exploration of soil bacterial communities (Agnelli et al. 2004). With the recent development in second-generation sequencing technologies, high-throughput sequencing has offered great advantages in determining soil microbial diversity and community composition (Kozich et al. 2013; Varma et al. 2018).

The rubber tree is an indigenous species of Amazon rainforests and serves as the sole source of natural rubber for the industry. The secondary laticifers located in the inner bark of rubber tree are the site for natural rubber biosynthesis and storage (Hao and Wu 2000). Latex, or the cytoplasm of laticifers, contains 20–40% rubber for natural rubber refinement. During natural rubber production, latex is collected by severing the laticifer rings every 2–3 days (Chao et al. 2017). The natural rubber yield per tree has significantly grown in the past 100 years, mainly due to variety selection and fertilizer application (Tang et al. 2013). Rubber plantations have become an important ecosystem in tropical areas such as Southeast Asia, Latin America, etc. (Dechner et al. 2018). Although soil bacterial communities of rubber plantations across seasons and chronosequence have been previously reported (Zhou et al. 2017; Lan et al. 2018), their response to different fertilizer treatments is poorly understood. Moreover, the physical properties of natural rubber make the degradation of waste rubber products difficult (Shah et al. 2012). Microbial degradation of natural rubber is an environmentally friendly way, and more than 100 rubber-degrading bacteria have been identified in the past decades (Luo et al. 2014). In the present study, we systematically investigated the effects of CF and OF–CF on natural rubber yield, soil properties, and soil bacterial community composition, and provided some recommendations for the use of OF to improve soil fertility in rubber plantations. Several possible rubber-degrading operational taxonomic units (OTUs) were further discussed.

## Materials and methods

### Experimental site and experimental design

The field experiment was located in the experimental farm of the Chinese Academy of Tropical Agricultural Sciences on Danzhou city, Hainan Province, China (19°51′51N; 109°55′63E). The experiment was conducted in a 4002-m^2^ area (3 m distance between trees and 5 m between rows, totaling 180 rubber trees). The rubber tree clone “CATAS73397” was planted in 2007 and was tapped in 2015. Beginning in Jan 2017, the experiment was established as a randomized complete block design with two treatment groups as follows: CF group subjected to CF treatment; and OF–CF group treated with OF plus CF. Each treatment contained three replicates. For CF treatment, the total CF (0.322 kg N, 0.378 kg phosphorus pentoxide [P_2_O_5_] 0.24 kg potassium oxide [K_2_O]) per tree was applied in April, July, and September at a proportion of 5:3:2, as per the flowering period of rubber tree (three times each year). For OF–CF treatment, 10 kg OF (0.187 kg N, 0.295 kg P_2_O_5_, 0.03 kg K_2_O) per tree was applied in January as basal fertilizer and the CF (0.135 kg N, 0.083 kg P_2_O_5_, 0.21 kg K_2_O) was used as the top dressing in April, July, and September at a proportion of 5:3:2. “The 4th element” CF (N + P_2_O_5_ + K2O ≥ 45%, 15-15-15) was produced by Stanley Agriculture Group Co., Ltd. (Shandong, China), while “Wo-Chen biological organic fertilizer” OF (N + P_2_O_5_ + K_2_O ≥ 5%; organic content ≥ 45%) was produced by Woyuan Organic Fertilizers Company (Shandong, China). The main source of OF came from animal manure.

### Soil samples collection

In December 2017, 180 soil cores (50 cm from the rubber tree trunk at a depth of 20 cm) were collected and categorized into six groups (two treatments, three replicates). Each soil sample was passed through a 2-mm mesh sieve before sampling. In each group, soil samples were pooled and split into two collections; one was used for the determination of soil properties, and other was stored at − 80 °C for soil microbiological high-throughput sequence analysis.

### Soil properties determination

#### Soil pH

A total of 10 g air-dried soil sample was added to 20 mL double-distilled water (ddH_2_O), and left still for 30 min. The supernatant was used for pH determination using a pH meter (FE28-Bio, Mettler-Toledo Sales International GmbH, Greifensee, Switzerland).

#### Soil organic carbon (SOC)

About 0.1 g air-dried soil sample and 0.1 g silver sulfate (AgSO_4_) were added into 5 mL potassium dichromate (K_2_Cr_2_O_7_)–sulphuric acid (H_2_SO_4_) solution (0.4 M), and treated at 200 °C in an oil bath for 5 min. The remaining K_2_Cr_2_O_7_ was titrated with iron sulfate (FeSO_4_). The content of SOC was calculated from the amount of K_2_Cr_2_O_7_ consumed (Li et al. 2009).

#### Soil total nitrogen (TN)

A total of 0.1 g air-dried soil sample was mixed with an accelerator (10 g potassium sulfate [K_2_SO_4_], 1 g copper sulfate [CuSO_4_], 0.1 g selenium [Se]), and boiled with 30 mL H_2_SO_4_ for 5 h. Nitrogen content in the digestion solution was determined by KjelMaster K-375 (BÜCHI Labortechnik AG, Flawil, Switzerland) (Wang et al. 2016b).

#### Available nitrate (AN)

In brief, 2 g air-dried soil sample was boiled with 10 mL calcium chloride (CaCl_2_, 0.01 M) for 16 h, and the AN content was determined using the BRAN + LUEBBE auto-analyzer (Bran + Luebbe GmbH, Norderstedt, Germany) (Mussa et al. 2009).

#### Available potassium (AK)

About 2.5 g air-dried soil sample was added into 50 mL sodium bicarbonate (NaHCO_3_, 0.5 M) and 20 mL supernatant was collected by centrifuge (12,000 rpm, 10 min). The supernatant was mixed with 5 mL molybdenum antimony reagent, and the AK content was determined using a PE Lambda 25 UV spectrophotometer (PerkinElmer, Waltham, USA) (Mengel et al. 1993).

#### Available phosphorus (AP)

Briefly, 5 g air-dried soil sample was mixed with 50 mL ammonium acetate (NH_4_OAc), and 20 mL of the supernatant was collected by centrifugation (12,000 rpm, 10 min) and used for AP determination using a Sherwood M410 flame photometer (Sherwood Scientific Ltd, Cambridge, UK) (Blake et al. 2003).

### Natural rubber yield characteristics analysis

The natural rubber content per sample was determined as previously described (Chao et al. 2015). In brief, 0.1 mL acetic acid was dropped into 1 g latex to obtain rubber coagula. The coagula were soaked in water for 2 h, dried overnight at 55 °C, and weighed. The natural rubber content was defined as follows:

$$ \frac{{{\text{Coagula weight }}\left( {\text{g}} \right)}}{{1\,{\text{g}}}} \times 100\% . $$

The latex yield (mL) is termed as the volume of latex collected by one tapping. The dry natural rubber yield (g) was determined as the product of natural rubber content (%) and latex yield (mL).

### Microbiological analysis

#### DNA extraction

A total of 0.5 g frozen soil of each group was used for the extraction of genomic DNA based on the manufacturer’s instructions in the Mo Bio Power Soil™ kit (Mo Bio, Carlsbad, CA, USA). The concentration and quality of DNA were examined by NanoDrop 2000 (Thermo Scientific Inc., Wilmington, DE, USA), and the integrity of the DNA was checked by 1.2% agarose gel electrophoresis.

#### 16S rRNA sequence

The bacterial 16S rRNA genes were amplified from soil genomic DNA using barcoded universal prokaryotic primers 515-forward (5′-GTG CCA GCM GCC GCG GTA A-3′) and 806-reverse (5′-GGA CTA CVS GGG TAT CTA AT-3′), designed against the V4 region of the bacterial 16S rRNA gene (Kozich et al. 2013). Polymerase chain reaction (PCR) was performed as follows: 95 °C for 3 min followed by 35 cycles of 95 °C for 45 s, 50 °C for 60 s and 72 °C for 90 s, as well as a final extension at 72 °C for 10 min. Each sample was amplified in triplicate, and equimolar amounts of amplicons were pooled for sequencing using the Illumina MiSeq platform (Allwegene Tech., Beijing, China).

#### Sequence accession numbers

The sequence information was deposited at the NCBI Sequence Read Archive (SRA) with the accession number SRP159519.

### Bioinformatic analyses of sequence data

Clean sequence data were obtained by removing the incorrect primer sequences as well as the sequences < 200 bp in length with homopolymers longer than six nucleotides, with quality scores below 20, and with ambiguous base calls. Clean data were clustered into OTUs at 97% sequence similarity using UCLUST (Edgar 2010). The bacterial taxonomic richness, diversity and evenness analysis were evaluated by MOTHUR, an open-source platform integrating multi-software (Schloss et al. 2009). The formula used for the calculation of Chao1 was defined as (Chao 1984):

$$ {\text{Chao1}} = {\text{Sobs}} + \frac{{n\left( {n1 - 1} \right)}}{{2\left( {n2 + 1} \right)}}. $$

The formulae employed for the calculation of Shannon index is defined as (Shannon 1948):

$$ H = - \mathop \sum \limits_{i = 1}^{s} \left( {p_{i} } \right)\left( {\ln p_{i} } \right). $$

The formulae utilized for the calculation of Simpson index is defined as (Simpson 1949):

$$ D = 1 - \mathop \sum \limits_{i = 1}^{s} p_{i}^{2} . $$

### Statistical analysis

The correlation among microbial phyla, soil physicochemical characteristics, and natural rubber yield was analyzed with redundancy analysis carried out using the “vegan” package of R (Nietomoreno et al. 2011). Statistical analysis was performed with SPSS Statistics 17.05 using the analysis of variance (ANOVA) based on independent-sample *t* test. The capital letter or ** represents *p* < 0.01, while the lower-case letter or * represents *p *< 0.05.

## Results and discussion

### The difference in soil physicochemical characteristics and natural rubber yield

The application of CF or OF–CF notably affected several key soil physicochemical characteristics (Table 1). Soil pH is an important factor determining the biochemical processes in soil (Stevens et al. 1998; Du et al. 2010). The over-application of CF causes soil acidification through the release of acidic organic decomposable compounds (Sial et al. 2019), while OF application relieves soil acidification due to the high levels of base cations (Ca^2+^, Mg^2+^, and K^+^) concentrations (Zeng et al. 2017). In comparison with CF treatment, OF–CF treatment increased the soil pH (Table 1). We propose that the high concentration of base cations released from OF may neutralize the acidic anions, and thus prevent soil acidification in rubber plantations. Moreover, the contents of TN, AN, AP, AK, and SOM increased in the soil treated with OF–CF (Table 1), hinting that OF application improves the properties of rubber plantation soil. Additionally, the rubber dry yield rose by 26.3% following OF–CF treatment (48.5 g/tree.tap) as compared with that observed following CF treatment (38.4 g/tree.tap) (Table 1). Thus, we suggest that the improvements in the properties of rubber plantation soil enhance the nutrient uptake efficiency of the rubber tree, thereby resulting in an increase in the yield of natural rubber, consistent with the results of previous studies with other crops (Martínez-Alcántara et al. 2016; Wang et al. 2016a).

**Table 1:** Soil properties of rubber plantations upon different fertilizer treatments

	CF	OF–CF
pH	4.39Ab	4.74Aa
SOM (g/kg)	15.01B	20.04A
TN (g/kg)	1.22B	1.82A
AN (mg/kg)	106.58B	180.02A
AP (mg/kg)	25.40B	30.27A
AK (mg/kg)	73.39Ab	108.93B
RDY (g/tree.tap)	38.40B	48.50A

The capital letter represents *p* < 0.01 while lower case represents *p* < 0.05. The same letter indicated no significant difference among groups

### Analysis of sequencing data and bacterial taxonomic richness

**Fig. 1:**
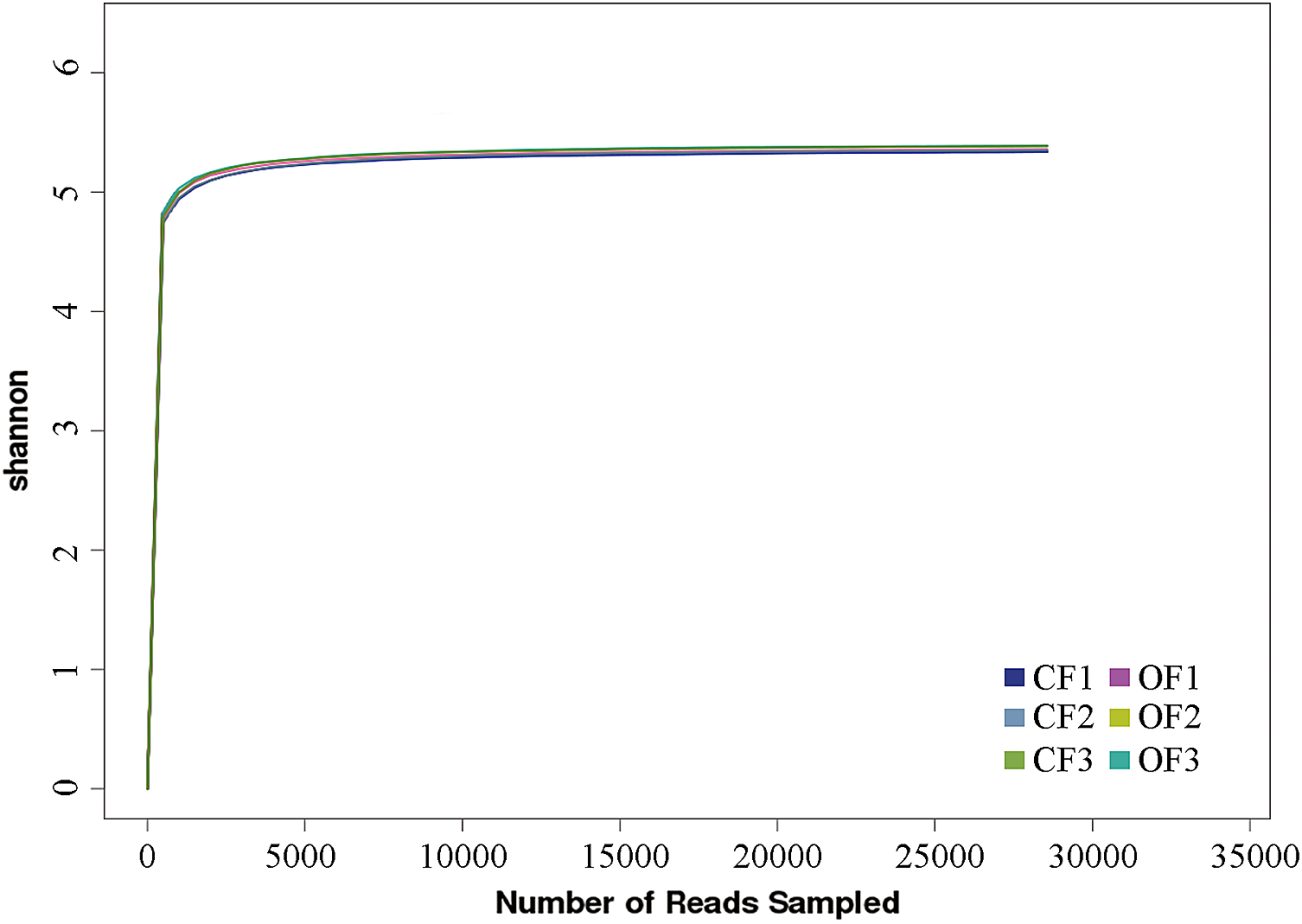
Shannon–Wiener curve of six samples

**Fig. 2:**
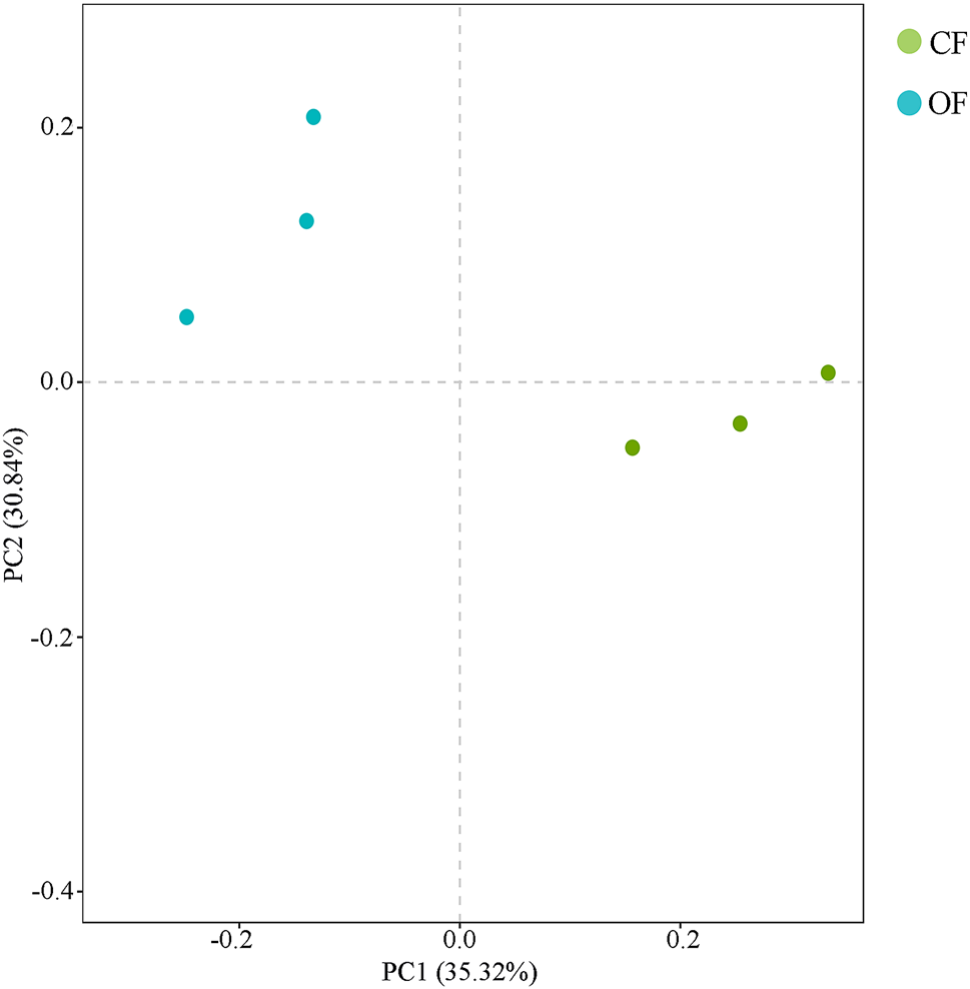
Principal component analysis of the soil bacterial communities in all six samples

**Fig. 3:**
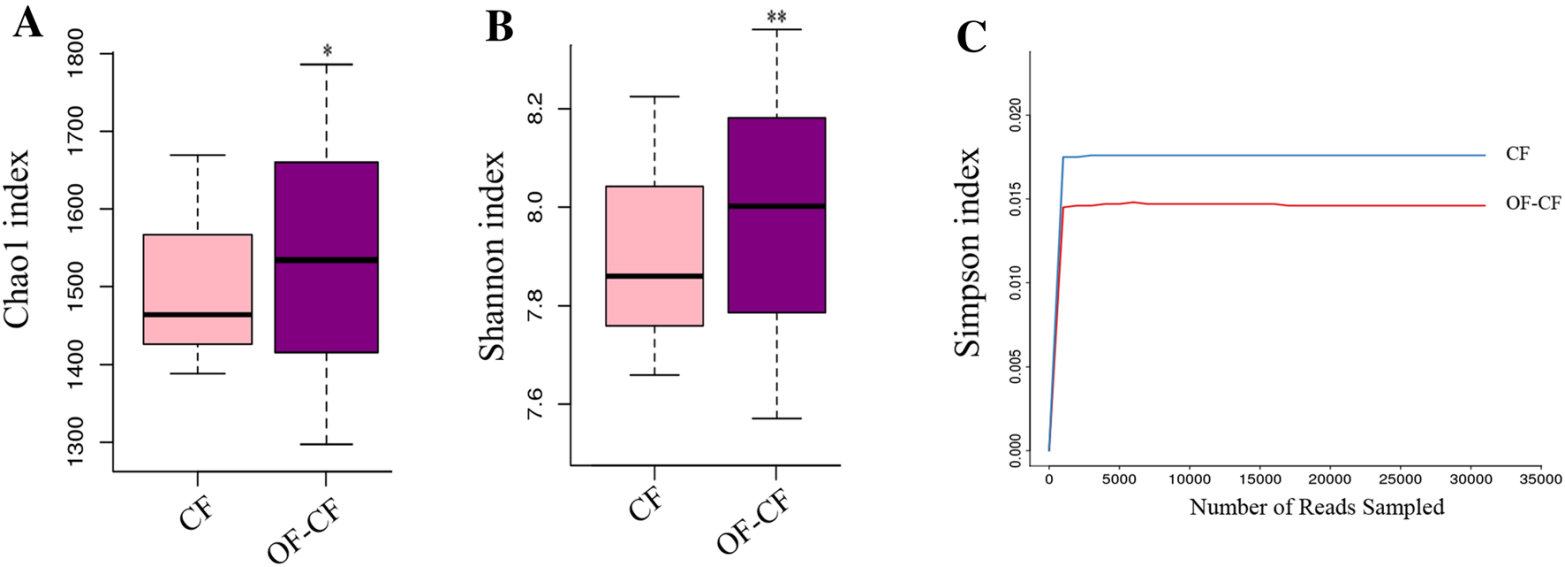
Chao1 (**a**), Shannon (**b**), and Simpson (**c**) indexes for the different treatments

Soil microbial organisms affect the function of the soil ecosystem and play an important role in the regulation of plant growth. We used the Illumina platform to sequence the 16S rRNA from six soil samples (three repetitions for each treatment). After the exclusion of low-quality tags, more than 50,000 clean tags were generated in each pool. The Shannon–Wiener curve showed that the sequencing data were large enough to reflect the information about the bacterial diversity in the samples (Fig. 1). At 97% similarity, a total of 1071 OTUs were obtained. From the total OTUs, 927 of these OTUs were identified from CF treatment group; 955 OTUs were detected from in OF–CF treatment group; while 916 OTUs were observed in both two treatment groups. The principal coordinate analysis (PCoA) of UniFrac distance matrices indicated that the differences in the microbiota of the two treatment (CF and OF–CF) groups (Fig. 2), suggesting that fertilizer application influences the community structure of the soil bacteria. Chao1, Shannon, and Simpson are the key indexes of bacterial *α*-diversity. In general, Chao1 represents the richness of bacterial communities (Peng et al. 2017). In Fig. 3a, we show that Chao1 was higher in the OF–CF treatment group (1573 ± 24) than that in the CF treatment group (1502 ± 36). With the exception of bacterial diversity, Shannon index and Simpson index can also reflect bacterial evenness (Yang et al. 2015). In the present study, Shannon index was higher in the OF–CF treatment group (7.90 ± 0.10 [CF] vs 8.05 ± 0.08 [OF–CF]) (Fig. 3b), while Simpson index was higher in the CF treatment group (0.0175 ± 0.0012 [CF] vs 0.0146 ± 0.0008 [OF–CF]) (Fig. 3c). Considering that Shannon index is negatively correlated with Simpson index, we thus deduce that OF application improves the richness, diversity, and evenness of bacterial communities possibly through soil pH renovation and nutrient content improvement (Liu et al. 2015; Wang et al. 2016a).

### Bacterial community composition and ecological significance of the selected groups

**Fig. 4:**
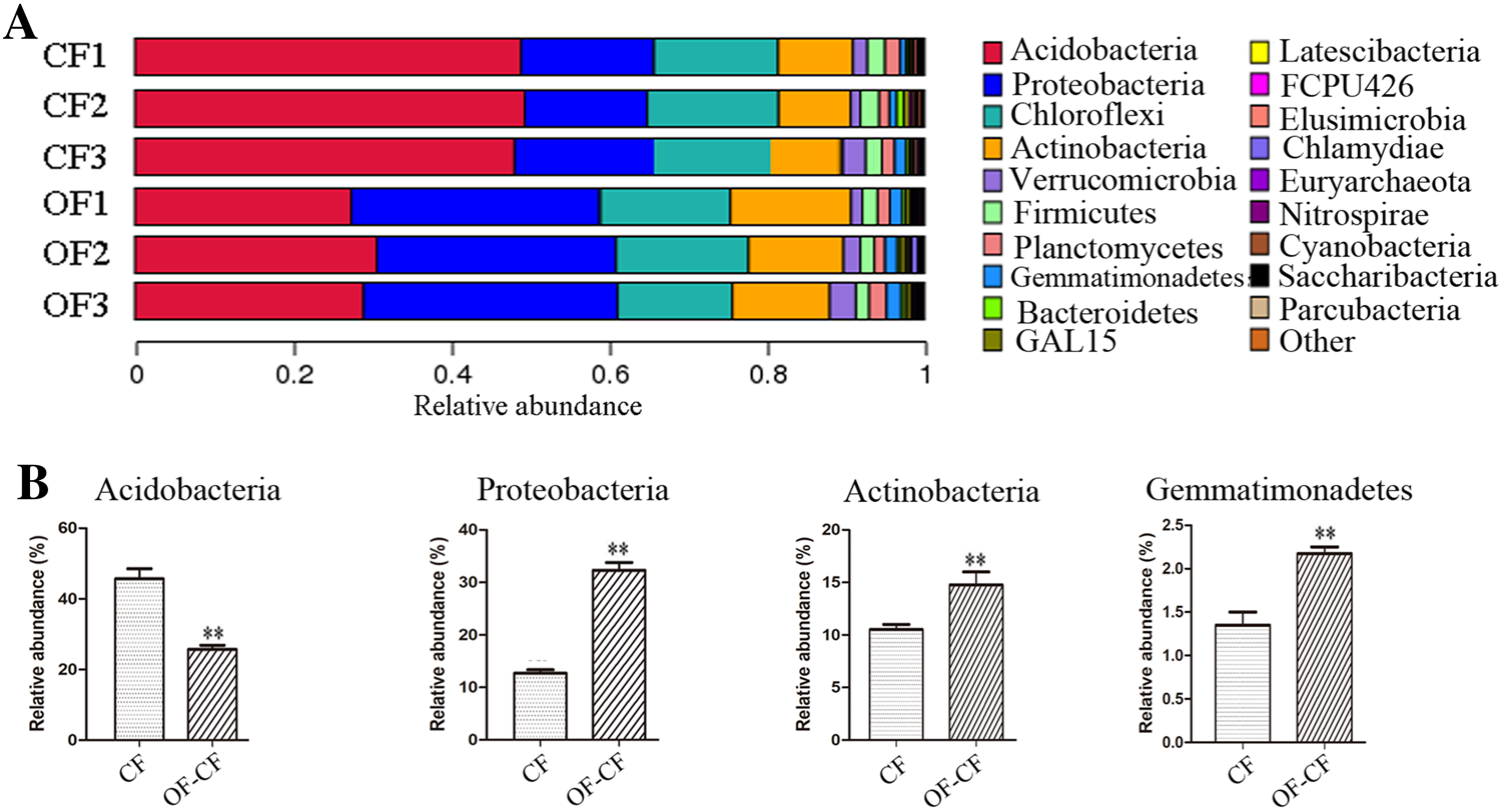
Relative abundance of soil bacterial phyla (**a**) and four phyla (**b**) under different fertilizers treatments. Average relative abundance was calculated from three replicates. The capital letter represents *p* < 0.01 while lower case represents *p* < 0.05. The same letter indicated no significant difference among groups

We grouped the 1071 OTUs into 23 phyla, 58 classes, 77 orders, 133 families, 134 genus, and 23 species (Table S1). At the phylum level, Acidobacteria (relative abundance 28.6–49.5%) was identified as the dominant phylum, followed by Proteobacteria (12.2–32.6%), Chloroflexi (15.3–25.0%), and Actinobacteria (8.6–16.4%). Verrucomicrobia (2.1–3.4%), Firmicutes (1.7–2.5%), Planctomycetes (1.3–2.1%), and Gemmatimonadetes (1.4–1.9%) were noted as minor phyla. The relative abundance of 11 phyla (Bacteroidetes, GAL15, Latescibacteria, FCPU426, Elusimicrobia, Chlamydiae, Euryarchaeota, Nitrospirae, Cyanobacteria, Saccharibacteria, Parcubacteria, and others) was below 1% (Fig. 4a). Interestingly, the top five phyla are the same as previously reported (Zhou et al. 2017; Lan et al. 2018), suggesting they are the dominant taxa in rubber plantations. At the species level, several OTUs are termed as “unidentified” (Table S1), indicating that most bacteria in rubber plantations have not been previously identified.

The change in the bacterial communities may reprogram soil properties (Chen et al. 2018). In the present study, we found that the relative abundances of four phyla were different among the two treatment groups (Fig. 4b). Acidobacteria is a group of oligotrophic bacteria found in nutrient-poor and highly acidic soil environments (Jones et al. 2009; Wang et al. 2016a). The abundance of Acidobacteria was higher following CF treatment than after OF treatment (*p *= 0.0015), consistent with the low pH value of the samples from CF treatment groups (Fig. 4b; Table 1). Actinobacteria are Gram-positive bacteria, while Proteobacteria and Gemmatimonadetes belong to Gram-negative bacteria. The relative abundances of these phyla were higher in the OF–CF treatment group than that in the CF treatment group (*p *= 0.0008, 0.0092, 0.0053 for Actinobacteria, Proteobacteria, Gemmatimonadetes, respectively) (Fig. 4b). Previous researches have shown that some taxa of Actinobacteria, Proteobacteria, and Gemmatimonadetes are beneficial for maintaining or improving soil fertility. Phosphorus (P)-solubilizing bacteria convert insoluble inorganic P into soluble forms, and play a crucial role in increasing the bioavailability of soil phosphates for plants (Adnan et al. 2017). Bello-Akinosho et al. (2016) identified that a strain belonging to Actinobacteria and two isolates belonging to Proteobacteria phyla displayed high phosphate solubilization index. Recently, Zeng et al. (2014) reported Gemmatimonadetes as a new phototrophic bacterial phylum, which plays a crucial role in the oxidation of organic compounds and fixation of N_2_ (Dubbs and Tabita 2004).

### Correlation analysis between bacterial taxa and physicochemical characteristics of the selected soil samples

**Fig. 5:**
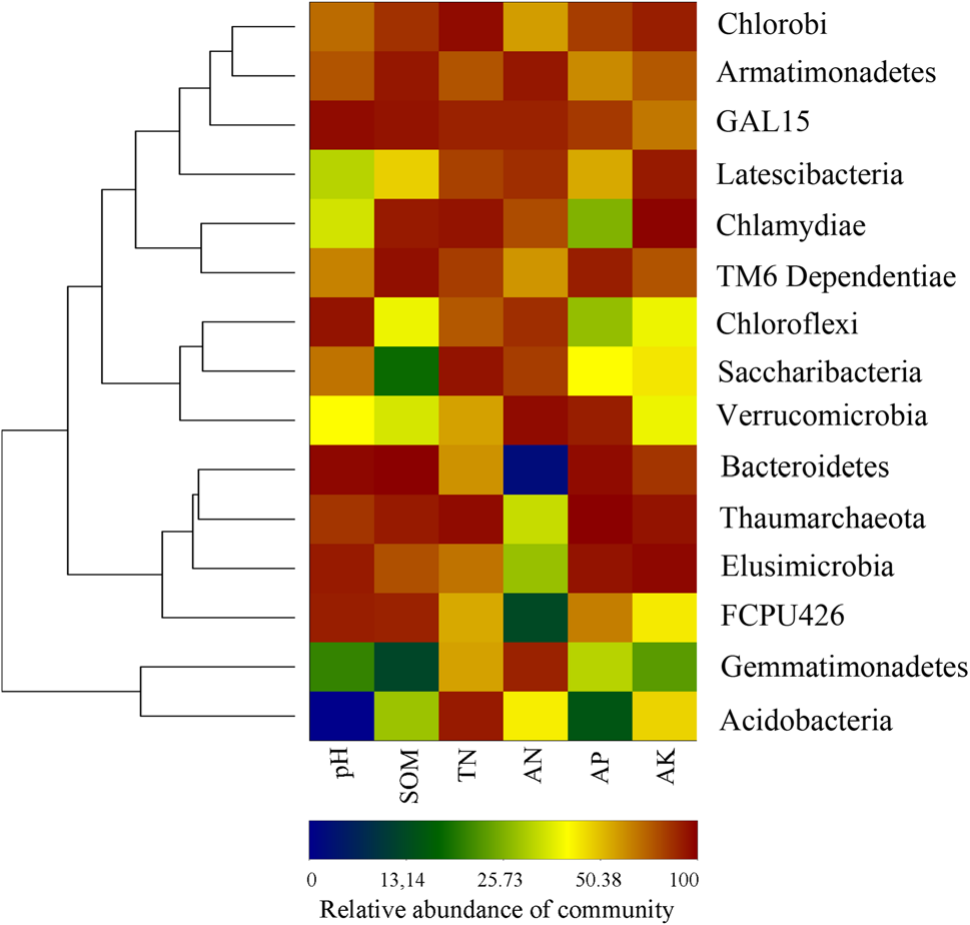
Correlation heat map between the bacterial phyla and the soil properties. Bar represented the relative abundance of community. Blue color showed negative correlation while red color showed positive correlation

We performed a correlation analysis between the bacterial taxa and soil physicochemical characteristics. Several phyla showed a strong correlation with soil properties (Fig. 5). Carbon mineralization rate is an important index of carbon availability in soil. By analysis of correlations between soil properties and bacterial taxa abundances, Fierer et al. (2007) reported that the abundances of Bacteroidetes were positively correlated with carbon mineralization rates. In the present study, Bacteroidetes showed a positive correlation with SOM (*r* = 0.992), an important carbon source for plant. Nitrogen fixation caused by microorganism is a key component of the global nitrogen cycle (Dang et al. 2013). The existence of *Ammonia monooxygenase A* gene is widespread in ammonia-oxidizing bacteria such as Thaumarchaeota (Dang et al. 2013). Wertz et al. (2012) sequenced the genome of Verrucomicrobia strain *Diplosphaera colitermitum*, and identified two nitrogen fixation genes: *nif* and *anf*. The present results showed Thaumarchaeota and Verrucomicrobia were found to exhibit a positive correlation with TN (*r *= 0.956) and AN (*r *= 0.960), respectively. As an ancient phylum, Thaumarchaeota possesses *Phn* gene encoding a phosphonate-specific uptake transporter (Dang et al. 2013). It was observed that Thaumarchaeota showed a positive correlation with AP (*r *= 0.996) in the present study. Evidence suggests that Elusimicrobia phylum plays a key role in nitrogen fixation (Zheng et al. 2016), but in our study, Elusimicrobia showed a positive correlation with AK (*r* = 0.972). Thus, some taxa within this phylum may regulate potassium transformation in the soil of rubber plantations.

### OTUs probably related to natural rubber degradation

In the past decades, several genera were found to be highly related to natural rubber degradation (Rose and Steinbüchel 2005). *Nocardia* sp. strain 835A is a well-studied rubber-degrading bacterium (Tsuchii et al. 1985). In the present study, we identified that OTU_506 belonged to *Nocardia* genus (Table S2). The species from *Streptomyces* genus have been frequently investigated for rubber biodegradation ability. Several *Streptomyces* spp. may shift rubber molecular mass in response to incubation with natural rubber latex after 10 weeks (Bode et al. 2000; Rose et al. 2004). In this study, we found that OTU_391 belonged to *Streptomyces* genus (Table S2). The rubber-degrading *Mycobacterium fortuitum* strain NF4 belongs to *Mycobacterium* genus. Linos et al. (2000) observed that *M. fortuitum* NF4 cells were directly embedded and merged into the rubber matrix after 1 week from inoculation. Our sequencing data showed that three OTUs (OTU_189, OTU_278, and OTU_1057) belonged to *Mycobacterium* genus (Table S2). Future work will involve isolation of these strains and verification of their roles in natural rubber degradation.

## Conclusion

In conclusion, the responses of soil properties and bacterial communities in rubber plantations to CF or OF–CF treatment were systematically investigated. The richness, diversity, and evenness of the bacterial community increased following OF–CF application. The relative abundances of several bacterial taxa were reprogrammed, and five bacterial taxa showed a strong correlation with the selected soil properties. Five OTUs were thought to be related to the biodegradation of natural rubber. The results listed here not only provide some recommendations for the understanding of the role of OF in improving soil fertility, but also contribute to the identification of rubber-degrading bacteria in rubber plantations.

## Electronic supplementary material

Below is the link to the electronic supplementary material.

Supplementary material 1 (DOC 72 kb). Categories of bacterial compositions at different levels

Supplementary material 2 (DOCX 14 kb). OTUs related to natural rubber degradation
