# Supplementary material for: Response of bacterial communities in rubber plantations to different fertilizer treatments
Source: 3 Biotech. 2019 Jul 4;9(8):293. doi: 10.1007/s13205-019-1821-6 (PMC6609652; doi:10.1007/s13205-019-1821-6)
Supplement: Supplementary file 1 — Supplementary material 1 (DOC 72 kb). Categories of bacterial compositions at different levels [file 13205_2019_1821_MOESM1_ESM.doc]

Table S1 Categories of bacterial compositions at different levels.

| **Phyla** | **Classes** | **Orders** | **Families** | **Genus** | **Species** |
| --- | --- | --- | --- | --- | --- |
| **Acidobacteria** | Acidobacteria (32); Blastocatellia (3); Holophagae (7); Solibacteres (22); Subgroup_13 (10); Subgroup_15 (1); Subgroup_17 (2); Subgroup_2 (20); Subgroup_5 (2); Subgroup_6 (18); | Acidobacteriales (32); Blastocatellales (3); Holophagales (3); Solibacterales (22); Subgroup_7 (4); unidentified (53) | Acidobacteriaceae_Subgroup_1 (32); Blastocatellaceae_Subgroup_4 (3); Holophagaceae (3); Solibacteraceae_Subgroup_3 (22); unidentified (57) | *11-24* (2); *Acidobacterium* (3); *Bryobacter* (12); *Candidatus_Koribacter* (11); *Granulicella* (1); *Holophaga* (2); *PAUC26f* (1); *Vicinamibacter* (2); *unidentified* (77) | *unidentified* (111) |
| **Actinobacteria** | Acidimicrobiia (40); Actinobacteria (54); TakashiAC-B11 (2); Thermoleophilia (21) | Acidimicrobiales (40); Catenulisporales (3); Corynebacteriales (5); Frankiales (19); Gaiellales (11); Kineosporiales (1); Micrococcales (3); Micromonosporales (5); Propionibacteriales (2); Pseudonocardiales (8); Solirubrobacterales (10); Streptomycetales (2); Streptosporangiales (6); unidentified (2) | 0319-6M6 (1); Acidimicrobiales_Incertae_Sedis (2); Acidothermaceae (17); Catenulisporaceae (1); Conexibacteraceae (1); Elev-16S-1332 (1); Frankiaceae (2); Gaiellaceae (1); Iamiaceae (1); Intrasporangiaceae (1); Kineosporiaceae (1); Micrococcaceae (2); Micromonosporaceae (5); Mycobacteriaceae (3); Nocardiaceae (2); Nocardioidaceae (2); Pseudonocardiaceae (8); Streptomycetaceae (2); Thermomonosporaceae (4); YNPFFP1 (2); unidentified (40) | *Aciditerrimonas* (1); *Acidothermus* (15); *Actinoallomurus* (1); *Actinobacterium_YJF2-33* (1); *Actinomadura* (2); *Actinospica* (2); *Amycolatopsis* (1); *Candidatus_Microthrix* (2); *Catenulispora* (1); *Conexibacter* (1); *Crossiella* (1); *Frankia* (1); *Gaiella* (1); *Iamia* (1); *Jatrophihabitans* (1); *Kribbella* (1); *Longispora* (1); *Luedemannella* (2); *Microbispora* (1); *Mycobacterium* (3); *Nocardia* (1); *Nocardioides* (1); *Pseudonocardia* (4); *Sinomonas* (1); *Smaragdicoccus* (1); *Streptomyces* (1); *unidentified* (45) | *Actinobacterium_YJF2-33* (1); *Thermomonosporaceae_bacterium_YE4-D4-16-CH2* (1); *unidentified* (90) |
| **Armatimonadetes** | Fimbriimonadia (1); Chthonomonadetes (1); Fimbriimonadia (2); unidentified (1) | Fimbriimonadales (1); Chthonomonadales (1); Fimbriimonadales (2); unidentified (1) | Fimbriimonadaceae (1); Fimbriimonadaceae (2); unidentified (2) | *unidentified* (5) | *unidentified* (5) |
| **Bacteroidetes** | Cytophagia (6); Flavobacteriia (1); Sphingobacteriia (26) | Cytophagales (6); Flavobacteriales (1); Sphingobacteriales (26) | Chitinophagaceae (12); CWT_CU03-E12 (4); Cytophagaceae (6); env.OPS_17 (3); Flavobacteriaceae (1); NS11-12_marine_group (1); Sphingobacteriaceae (5); unidentified (1) | *Cytophaga* (1); *Dinghuibacter* (1); *Flavisolibacter* (2); *Flavobacterium* (1); *Mucilaginibacter* (5); *Niabella* (1); *Sporocytophaga* (1); *unidentified* (18) | *Cytophaga_aurantiaca* (1); *Dinghuibacter_silviterrae* (1); *unidentified* (27) |
| **Chlamydiae** | Chlamydiae (30) | Chlamydiales (30) | Chlamydiaceae (1); cvE6 (2); Parachlamydiaceae (12); Simkaniaceae (14) | *Candidatus_Protochlamydi*a (3); *Neochlamydia* (6); *unidentified* (13) | *Chlamydiae_bacterium_Ga0074140* (1); *unidentified* (20) |
| **Chlorobi** | Chlorobia (4) | Chlorobiales (4) | OPB56 (4) | *unidentified* (4) | *unidentified* (4) |
| **Chloroflexi** | Anaerolineae (13); Elev-1554 (4); JG30-KF-CM66 (16); JG37-AG-4 (21); KD4-96 (1); Ktedonobacteria (198); S085 (2); SBR2076 (3); Thermomicrobia (7); TK10 (13) | Anaerolineales (13); B10-SB3A (3); B12-WMSP1 (15); bacterium_Ellin6543 (4); C0119 (2); JG30-KF-AS9 (50); JG30-KF-CM45 (7); Ktedonobacterales (102); Thermogemmatisporales (18); unidentified (53) | 1921-2 (18); 1921-2 (4); 1959-1 (3); Anaerolineaceae (13); BacC-u-018 (3); bacterium_Ellin6537 (6); FCPS473 (11); G12-WMSP1 (4); HSB_OF53-F07 (30); Ktedonobacteraceae (6); Thermosporotrichaceae (12); unidentified (128) | *bacterium_Ellin6537* (6); *Ktedonobacter* (1); *Thermosporothrix* (3); *unidentified* (221) | *bacterium_Ellin6543* (6); *unidentified* (225) |
| **Cyanobacteria** | Chloroplast (3); Melainabacteria (4) | Bromus_tectorum (1); Obscuribacterales (4) | Bromus_tectorum (1); unidentified (4) | *Bromus_tectorum* (1); *unidentified* (4) | *Bromus_tectorum* (1); *unidentified* (4) |
| **Elusimicrobia** | Elusimicrobia (18) | FCPU453 (1); Lineage_IIa (7); Lineage_IIb (1); Lineage_IIc (2); Lineage_IV (5) | unidentified (16) | *unidentified* (16) | *unidentified* (16) |
| **Euryarchaeota** | Thermoplasmata (10) | Thermoplasmatales (10) | Terrestrial_Miscellaneous_GpTMEG (9); Marine_Group_II (1) | *unidentified* (10) | *unidentified* (10) |
| **FCPU426** | unidentified (4) | unidentified (4) | unidentified (4) | *unidentified* (4) | *unidentified* (4) |
| **Firmicutes** | Bacilli (14); Clostridia (16); Erysipelotrichia (1) | Bacillales (14); Clostridiales (4); Erysipelotrichales (1); Halanaerobiales (9) | Alicyclobacillaceae (9); Bacillaceae (6); Clostridiaceae_1 (2); Erysipelotrichaceae (1); ODP1230B8.23 (9); Paenibacillaceae (2); Peptostreptococcaceae (1); Ruminococcaceae (1); Sporolactobacillaceae (1) | *Alicyclobacillus* (1); *Bacillus* (6); *Clostridium_sensu_stricto_3* (2); *Cohnella* (1); *Paenibacillus* (1); *Pullulanibacillus* (1); *Romboutsia* (1); *Ruminiclostridium_1* (1); *Tumebacillus* (3); *unidentified* (10) | *Bacillus_aryabhattai* (1); *Bacillus_circulans* (1); *Bacillus_drentensis* (1); *Bacillus_sp._BA315*(1); *Mycoplasmataceae*_b*acterium_RC_NB112A* (1); *Streptomyces_sp._KP17* (1); *unidentified* (20) |
| **GAL15** | unidentified (3) | unidentified (3) | unidentified (3) | *unidentified* (3) | *unidentified* (3) |
| **Gemmatimonadetes** | Gemmatimonadetes (36); S0134_terrestrial_group (1) | Gemmatimonadales(36); unidentified (1) | Gemmatimonadaceae (36); unidentified (1) | *Gemmatimonas* (18); *unidentified* (14) | *unidentified* (32) |
| **Latescibacteria** | unidentified (1) | unidentified (1) | unidentified (1) | *unidentified* (1) | *unidentified* (1) |
| **Nitrospirae** | Nitrospira (5) | Nitrospirales (5) | Nitrospiraceae (3); 0319-6A21 (1); unidentified (1) | *Nitrospira* (3); *unidentified* (2) | *unidentified* (5) |
| **Parcubacteria** | Candidatus_Azambacteria (3); unidentified (3) | Parcubacteria_group_bacterium_GW2011_GWA1_60_11 (2); unidentified (4) | Parcubacteria_group_bacterium_GW2011_GWA1_60_11 (2); unidentified (4) | *Parcubacteria_group_bacterium_GW2011_GWA1_60_11* (2); *unidentified* (4) | *Parcubacteria_group_bacterium_GW2011_GWA1_60_11* (2); *unidentified* (4) |
| **Planctomycetes** | BD7-11 (2); OM190 (1); Phycisphaerae (6); Pla4_lineage (2); Planctomycetacia (69) | Phycisphaerales (2); Planctomycetales (69); Tepidisphaerales (4); unidentified (5) | Phycisphaeraceae (2); Planctomycetaceae (69); Tepidisphaeraceae (4); unidentified (5) | *Isosphaera* (2); *Phycisphaera* (1); *Singulisphaera* (10); *unidentified* (57) | *Planctomycetia_bacterium_WSF3-27* (1); *unidentified* (70) |
| **Proteobacteria** | Alphaproteobacteria (116); Betaproteobacteria (29); Deltaproteobacteria (58); Gammaproteobacteria (70) | Bdellovibrionales (1); Burkholderiales (16); Caulobacterales (10); Desulfurellales (14); Enterobacteriales (1); HTA4 (3); Legionellales (29); Myxococcales (39); Neisseriales (2); Nitrosomonadales (6); Oligoflexales (3); Pseudomonadales (3); Rhizobiales (36); Rhodocyclales (1); Rhodospirillales (56); Rickettsiales (8); SAR324_cladeMarine_group_B (1); SC-I-84 (3); Sphingomonadales (5); X35 (1); Xanthomonadales (26) | 0319-6G20 (3); 27F-1492R (1); Acetobacteraceae (14); Alcaligenaceae (1); alphaI_cluster (1); Archangiaceae (3); Aurantimonadaceae (1); Bdellovibrionaceae (1); Beijerinckiaceae (2); BIrii41 (2); Blfdi19 (3); Bradyrhizobiaceae (3); Burkholderiaceae (7); Candidatus_Nitrosoarchaeum_limnia_SFB1 (1); Caulobacteraceae (8); Comamonadaceae (6); Coxiellaceae (26); DA111 (29); Desulfurellaceae (14); Enterobacteriaceae (1); Haliangiaceae (8); Holosporaceae (3); Hyphomicrobiaceae (4); Hyphomonadaceae (2); I-10 (1); JG37-AG-20 (1); KD3-10 (1); KF-JG30-B3 (1); Legionellaceae (3); LWSR-14 (1); Methylobacteriaceae (1); Mitochondria (2); mle1-27 (1); Moraxellaceae (3); Neisseriaceae (2); Nevskiaceae (2); Nitrosomonadaceae (6); Oxalobacteraceae (2); P3OB-42 (1); Phaselicystidaceae (3); Polyangiaceae (9); Rhizobiaceae (2); Rhizobiales_Incertae_Sedis (10); Rhodobiaceae (1); Rhodocyclaceae (1); Rhodospirillaceae (6); Rhodospirillales_Incertae_Sedis (2); Roseiarcaceae (2); Sandaracinaceae (4); SM2D12 (2); Solimonadaceae (1); Sphingomonadaceae (4); Xanthobacteraceae (6); Xanthomonadaceae (8); Xanthomonadales_Incertae_Sedis (12); unidentified (9) | *Acidibacter* (10); *Acidicaldus* (3); *Acidiphilium* (2); *Acidisoma* (1); *Acidisphaera* (1); *Anaeromyxobacter* (3); *Aquabacterium* (1); *Aquicella* (24); *Asticcacaulis* (1); *Aureimonas* (1); *Bauldia* (2); *Bdellovibrio* (1); *Blastochloris* (1); *Bosea* (1); *Bradyrhizobium* (1); *Burkholderia-Paraburkholderia* (5); *Candidatus_Nitrosoarchaeum_limnia_SFB1* (1); *Coxiella* (2); *Cupriavidus* (1); *Dyella* (2); *H16* (14); *Haliangium* (8); *Hyphomicrobium* (1); *Inquilinus* (1); *Labrys* (1); *Legionella* (3); *marine_metagenome* (1); *Mesorhizobium* (1); *Methylobacterium* (1); *Methylovirgula* (1); *Mizugakiibacter* (1); *Nevskia* (2); *Nitrosospira* (1); *Nordella* (1); *Pedomicrobium* (1); *Perlucidibaca* (2); *Phaeospirillum* (1); *Phaselicystis* (2); *Phenylobacterium* (3); *Phreatobacter* (1); *Polycyclovorans* (1); *Pseudolabrys* (1); *Ralstonia* (1); *Raoultella* (3); *Rhizobium* (2); *Rhizomicrobium* (6); *Rhodanobacter* (3); *Rhodomicrobium*(1); *Rhodovastum* (1); *Roseiarcus* (2); *Rudaea* (1); *Sandaracinus* (1); *Sorangium* (9); *Sphingomonas* (2); *Sphingopyxis* (1); *Spongospora_subterranea* (1); *Stenotrophomonas* (1); *Uliginosibacterium* (1); *Variovorax* (1); *Vogesella* (1); *Woodsholea* (2); *unidentified* (80) | *Candidatus_Nitrosoarchaeum_limnia_SFB*1 (1); *Crenobacter_luteus* (1); *marine_metagenome* (1); *Rhizobium_etli* (1); *Spongospora_subterranea* (1); *Stenotrophomonas*_*rhizophila* (1); *unidentified* (221) |
| **Saccharibacteria** | unidentified (8) | unidentified (8) | unidentified (8) | *unidentified* (8) | *unidentified* (8) |
| **Thaumarchaeota** | FHMa11_terrestrial_group (1) | unidentified (1) | unidentified (1) | *unidentified* (1) | *unidentified* (1) |
| **TM6_Dependentiae** | unidentified (6) | unidentified (6) | unidentified (6) | *unidentified* (6) | *unidentified* (6) |
| **Verrucomicrobia** | OPB35_soil_group (20); Opitutae (3); S-BQ2-57_soil_group (1); Spartobacteria (12) | Chthoniobacterales (12); Opitutales (3); unidentified (18) | Chthoniobacteraceae (2); DA101_soil_group (7); Opitutaceae (3); Xiphinematobacteraceae (3); unidentified (18) | *Candidatus_Xiphinematobacter* (3); *Chthoniobacter* (2); *Opitutus* (3); *unidentified* (25) | *unidentified* (33) |

Note: Number in bracket showed the OTUs number.
