# Supplementary material for: Response of bacterial communities in rubber plantations to different fertilizer treatments
Source: 3 Biotech. 2019 Jul 4;9(8):293. doi: 10.1007/s13205-019-1821-6 (PMC6609652; doi:10.1007/s13205-019-1821-6)
Supplement: Supplementary file 2 — Supplementary material 2 (DOCX 14 kb). OTUs related to natural rubber degradation [file 13205_2019_1821_MOESM2_ESM.docx]

Table S2 OTUs related to natural rubber degradation.

| OUT ID | Treatment^1^ | | Taxonomy^2^ |
| --- | --- | --- | --- |
|  | CF | OF-CF |  |
| OTU_506 | 17 | 18 | k__Bacteria; p__Actinobacteria; c__Actinobacteria; o__Corynebacteriales; f__Nocardiaceae; g__*Nocardia*; s__*unidentified* |
| OTU_391 | 39 | 42 | k__Bacteria; p__Actinobacteria; c__Actinobacteria; o__Streptomycetales; f__Streptomycetaceae; g__*Streptomyces*; s__*unidentified* |
| OTU_189 | 44 | 46 | k__Bacteria; p__Actinobacteria; c__Actinobacteria; o__Corynebacteriales; f__Mycobacteriaceae; g__*Mycobacterium*; s__*unidentified* |
| OTU_278 | 19 | 22 | k__Bacteria; p__Actinobacteria; c__Actinobacteria; o__Corynebacteriales; f__Mycobacteriaceae; g__*Mycobacterium*; s__*unidentified* |
| OTU_1057 | 3 | 2 | k__Bacteria; p__Actinobacteria; c__Actinobacteria; o__Corynebacteriales; f__Mycobacteriaceae; g__*Mycobacterium*; s__*unidentified* |

Note: 1. Arabic number represented sequence counts in each sample. 2. Taxonomic pedigree information. K, kingdom; p, phylum; c, class; o, order; f, family; g, genus; s, species.
